# Two new species of *Bryophaenocladius* Thienemann, 1934 (Diptera, Chironomidae) from China

**DOI:** 10.3897/zookeys.208.3378

**Published:** 2012-07-17

**Authors:** Xiaolong Lin, Xin Qi, Xinhua Wang

**Affiliations:** 1 College of Life Science, Nankai University, Tianjin 300071, China; 2 College of Life Science, Taizhou University, Taizhou, Zhejiang 318000, China

**Keywords:** Chironomidae, *Bryophaenocladius*, new species, key, China

## Abstract

Two new species of *Bryophaenocladius* Thienemann, 1934, *Bryophaenocladius mucronatus* **sp. n.** and *Bryophaenocladius parictericus* **sp. n.** are described and illustrated as males. A key to male imagines of the genus from China is presented.

## Introduction

The genus *Bryophaenocladius* was erected by Thienemann in 1934 with *Orthocladius muscicola* Kieffer, 1906 as type species. To date, more than 100 species have been recorded all over the world (Andersen and Schnell 2000, Ashe and Cranston 1990, Chaudhuri et al. 2001, Du and Wang 2010, Du et al. 2011, Freeman and Cranston 1980, Liu and Wang 2005, Makarchenko and Makarchenko 2006, 2009, 2011, Sæther 1973, Sæther et al. 2000, Sasa and Kikuchi 1995, Spies and Reiss 1996, Strenzke 1957, Wang 2000, Wang et al. 2001, 2004, 2006, Yamamoto 2004). So far 7 species of the genus were recorded in China, namely *Bryophaenocladius cuneiformis* Armitage, 1987, *Bryophaenocladius parimberbus* Du and Wang, 2010, *Bryophaenocladius propinquus* (Brundin, 1947), *Bryophaenocladius scanicus* (Brundin, 1947), *Bryophaenocladius vernalis* (Goetghebuer, 1921), *Bryophaenocladius wufengensis* Du and Wang, 2010, and *Bryophaenocladius xinglongensis* Du and Wang, 2010.

The adult males of most *Bryophaenocladius* species can be recognized by strong and decumbent acrostichals beginning close to antepronotum; wing membrane without setae, but with coarse punctation visible at 40x magnification, squama with one to several setae; tibial spurs strongly developed, with well developed, but not divergent lateral denticles; hind tibial comb well developed; sensilla chaetica absent; tergite IX distinctive, with strongly pigmented, semi-circular band running around posterior margin; anal point projecting from setose area, large, semicircular to triangular; virga consisting of simple spines; gonostylus often distinctly broadened, strong megaseta (Cranston et al. 1989). However, there are exceptions to nearly all of these diagnostic characters. *Bryophaenocladius psilacrus* Sæther is lacking acrostichals (Sæther 1982). Several species with bare squama (Andersen and Schnell 2000). The tibial spurs may be essentially without lateral denticles as in most Afrotropical species (Wang et al. 2001) and thus differ from the typical condition with lateral denticles separated but not as much as in *Chaetocladius* Kieffer. Tergite IX and the anal point may deviate from the typical form and it is the association of those species which are most in doubt such as *Bryophaenocladius productus* (Freeman, 1953) (Sæther 1973).

**Figure 1.**
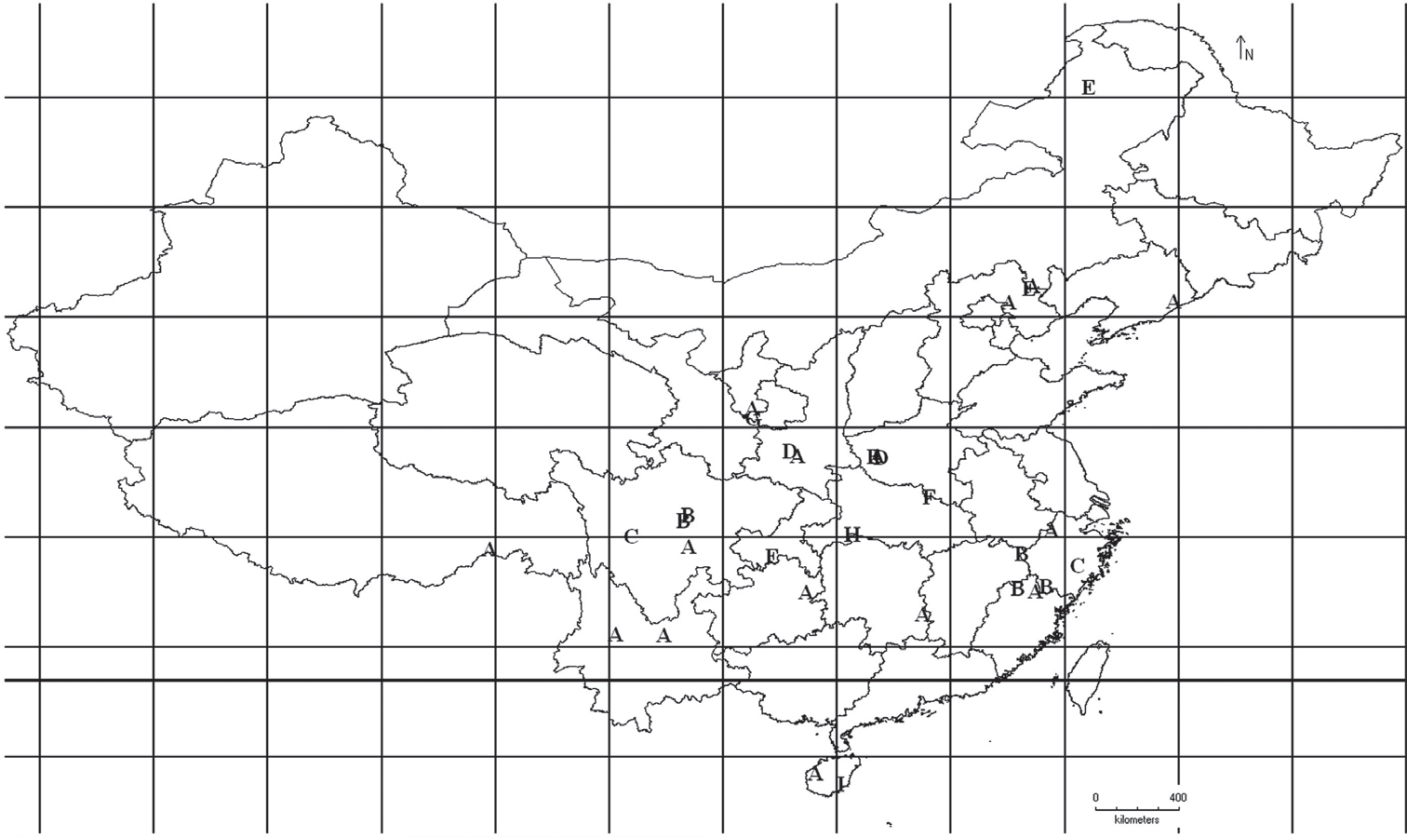
Distribution in China for the genus *Bryophaenocladius* **A** *Bryophaenocladius cuneiformis* Armitage, 1987 **B** *Bryophaenocladius mucronatus* sp. n. **C** *Bryophaenocladius parictericus* sp. n. **D** *Bryophaenocladius parimberbus* Du & Wang, 2010 **E** *Bryophaenocladius propinquus* (Brundin, 1947) **F** *Bryophaenocladius scanicus* (Brundin, 1947). **G** *Bryophaenocladius vernalis* (Goetghebuer, 1921) **H** *Bryophaenocladius wufengensis* Du & Wang, 2010 **I** *Bryophaenocladius xinglongensis* Du & Wang, 2010.

After examinzing the type specimen of *Bryophaenocladius bicolor* Wang, Sæther & Andersen, 2001 and the specimens of *Bryophaenocladius ictericus* (Meigen, 1830) collected from Canada, China and Sweden, two new species from oriental China are described. A key to male imagines of *Bryophaenocladius* from China and a distribution map of genus *Bryophaenocladius* in China is presented (Fig. 1).

## Materials and methods

The morphological nomenclature follows Sæther (1980) and the abbreviations of parts measured follow Qi et al. (2012). The material examined was mounted on slides, following the procedure outlined by Sæther (1969). Measurements are given as ranges followed by the mean, when three or more specimens are measured, followed by the number of specimens measured (n) in parentheses. Examined specimens in this study are deposited in the College of Life Science, Nankai University, China and College of Life Science, Taizhou University, China.

### Key to male imagines of *Bryophaenocladius* from China

**Table:** 

1	Third palpomere with apical projection	2
–	Third palpomere without apical projection	4
2	Squama with setae; AR>1.0	3
–	Squama bare; AR<1.0	*Bryophaenocladius parictericus* sp. n.
3	Inferior volsella unobvious	*Bryophaenocladius xinglongensis* Du & Wang, 2010
–	Inferior volsella obvious	*Bryophaenocladius cuneiformis* Armitage, 1987
4	Squama bare	5
–	Squama setose	6
5	Crista dorsalis absent; inferior volsella obvious	*Bryophaenocladius vernalis* (Goetghebuer, 1921)
–	Crista dorsalis present; inferior volsella unobvious	*Bryophaenocladius parimberbus* Du & Wang, 2010
6	Anal point broad	7
–	Anal point slender	8
7	Inferior volsella finger-shaped	*Bryophaenocladius propinquus* (Brundin, 1947)
–	Inferior volsella almost rectangular	*Bryophaenocladius scanicus* (Brundin, 1947)
8	Pseudospurs present on ta_1_, ta_2_ of mid and hind legs	*Bryophaenocladius mucronatus* sp. n.
–	Pseudospurs absent	*Bryophaenocladius wufengensis* Du & Wang, 2010

## Taxonomy

### Bryophaenocladius mucronatus sp. n.

urn:lsid:zoobank.org:act:09174531-D113-4061-B288-6627445DFCAF

http://species-id.net/wiki/Bryophaenocladius_mucronatus

Figures 2–4

#### Diagnosis.

The male imago can be distinguished from known species of the genus by the following combination of characters: third palpomere without apical digitiform projection; squama with 1–7, 4 setae; pseudospurs present on ta_1_ and ta_2_ of mid and hind legs; anal point hyaline, slender with pointed apex; tergite IX columnar; inferior volsella thumb-shaped, with 0–5, 3 setae.

#### Description.

Male imago (n = 29). Total length 2.20–3.00, 2.51 mm. Wing length 1.33–1.76, 1.55 mm. Total length/wing length 1.43–1.90, 1.65. Wing length/length of profemur 2.50–3.34, 2.75.

Coloration.Dark brown.

Head. AR 1.13–1.43, 1.26. Ultimate flagellomere 415–455, 430 μm long. Temporal setae 7–11, 9 including 2–4, 3 inner verticals; 4–6, 5 outer verticals and 1–2, 2 postorbitals. Clypeus with 2–5, 3 setae. Tentorium 105–150, 130 μm long, 18–25, 20 μm wide. Stipes 105–110, 108 μm long, 7–10, 8 μm wide. Palpomere lengths (in μm): 20–50, 35; 30–95, 47; 55–110, 80; 60–100, 80; 100–125, 113. L: 5^th^/3^rd^ 1.40–1.82, 1.56. Third palpomere without apical digitiform projection.

**Figures 2–4.**
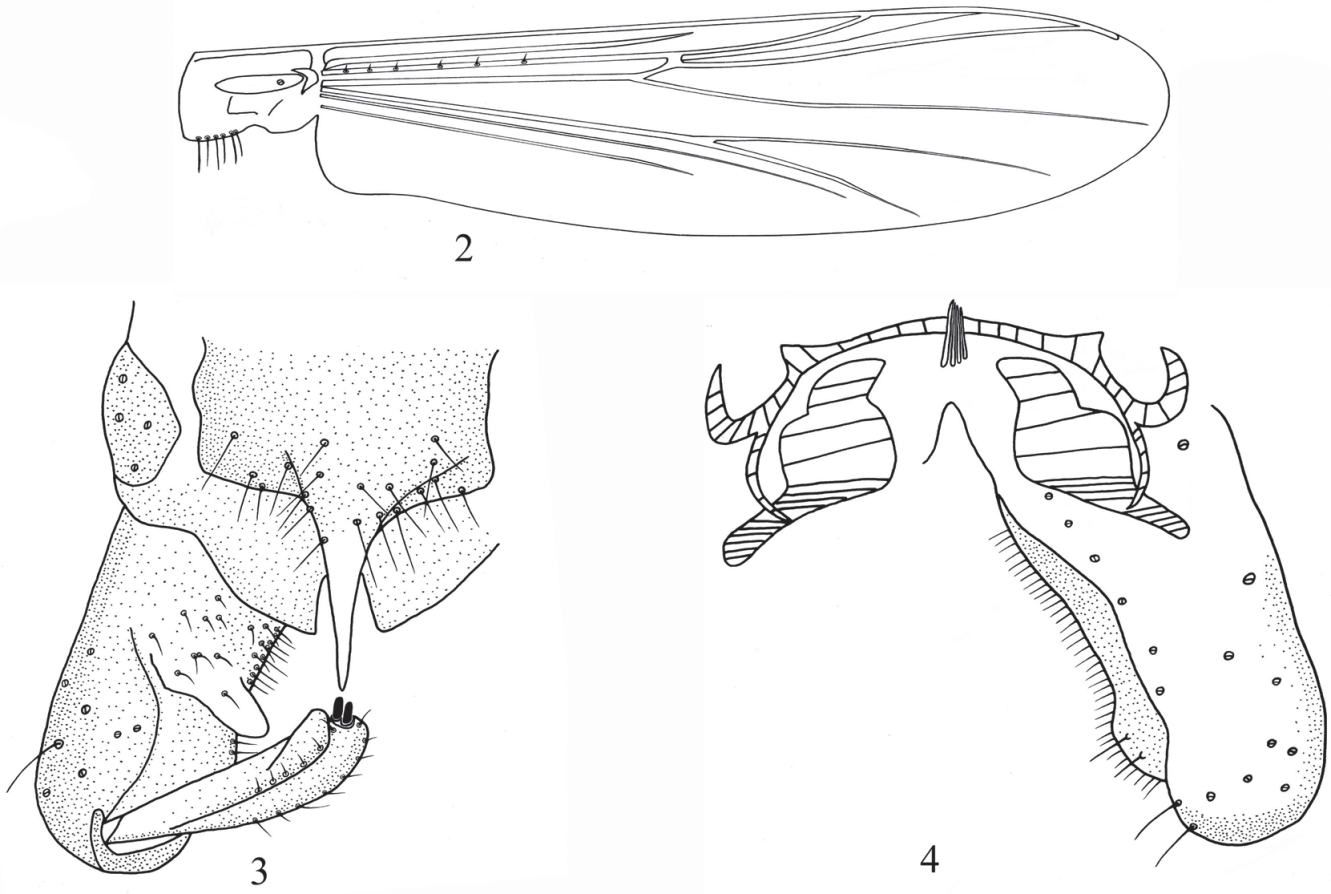
*Bryophaenocladius mucronatus* sp. n. **2** wing **3** hypopygium (dorsal view) **4** hypopygium (ventral view).

Wing (Fig. 2). Anal lobe developed. Coarse punctation easily visible at 40x magnification. VR 1.16–1.33, 1.26. Costa extension 40–63, 48 μm long. Brachiolum with 1–3, 2 setae. R with 3–6, 4 setae; R_4+5_ with 0–1, 0 seta. Remaining veins bare. Squama with 1–7, 4 setae.

Thorax. Antepronotum with 3–8, 4 lateral setae. Dorsocentrals 5–13, 9; acrostichals 3–10, 7; prealars 2–5, 3. Scutellum with 2–8, 4 setae.

Legs. Spur of fore tibia 16–65, 45 μm long; spurs of mid tibia 20–40, 33 μm and 12–27, 20 μm long; spurs of hind tibia 42–58, 50 μm and 11–40, 23 μm long. Lateral denticles appressed to main shaft. Hind tibial comb with 6–16, 13 spines. Pseudospurs present on ta_1_ and ta_2_ of mid and hind legs, 18–23, 20 μm long. Width at apex of fore tibia 23–38, 30 mm, of mid tibia 25–35, 27 mm, of hind tibia 30–40, 35 mm. Lengths (in μm) and proportions of legsas in Table 1.

**Table 1.** Lengths (in μm) and proportions of legs of *Bryophaenocladius mucronatus* sp. n.

	_P_1	_P_2	_P_3
fe	500–594, 553	588–650, 622	580–690, 643
ti	620–783, 723	570–704, 661	648–810, 765
ta_1_	370–450, 415	240–324, 288	300–450, 415
ta_2_	220–270, 240	140–190, 170	160–250, 220
ta_3_	160–200, 180	105–135, 123	135–200, 173
ta_4_	105–130, 118	60–90, 75	80–110, 95
ta_5_	80–100, 86	60–95, 80	75–100, 88
LR	0.52–0.63, 0.57	0.42–0.48, 0.45	0.46–0.59, 0.55
BV	2.59–2.62, 2.61	3.37–3.57, 3.47	3.13–3.28, 3.19
SV	2.96–3.10, 3.03	4.32–4.58, 4.47	3.16–3.51, 3.31
BR	2.17–2.86, 2.41	2.22–3.00, 2.47	3.33–4.35, 3.81

Hypopygium (Figs 3–4). Anal point hyaline, slender, with pointed apex, 45–90, 70 μm long, 25–35, 30 μm wide. Anal point length/width: 2.14–2.71, 2.45. Tergite IX columnar, with 10–22, 15 setae, laterosernite IX with 4–8, 6 setae. Phallapodeme 45–85, 70 μm long. Transverse sternapodeme arcuate with developed oral projection, 68–100, 88 μm long. Gonocoxite 175–212, 190 μm long. Gonostylus 68–100, 87 μm long with 1–2, 1 megaseta, 8–13, 10 μm long. Crista dorsalis low. Inferior volsella thumb-shaped, 23–35, 27 μm long, with 0–5, 3 setae. Virga 10–25, 16 μm long, composed of 1–9, 5 spines. HR 1.95–2.36, 2.12. HV 2.59–3.00, 2.71.

#### Type materials.

Holotype: ♂ (BDN. I4B20), China, Zhejiang Province: Quzhou City, Kaihua County, Gutian Mountain, 29°14'35"N, 118°06'41"E, 18.iv.2011, Lin XL, sweeping net. Paratypes (28♂♂): 2♂♂, as holotype; 1♂, Zhejiang Province, Lishui City, Qinyuan County, 27°45'08"N, 119°12'26"E, 15.iv.1994, Wu H, sweeping net; Fujian Province: 11♂♂, Wuyi Mountain, 27°38'22"N, 117°56'56"E, 26.iv.1993, Wang XH, sweeping net; Sichuan Province: 7♂♂, Wenchuan County, 30°59'27"N, 103°26'44"E, 14.vii.1987, Li XZ, sweeping net; 7♂♂, Wolong National Nature Reserve, 30°45'23"N, 103°13'55"E, 27.vii.1987, Li XZ, sweeping net.

#### Etymology.

The species name is from Latin *mucronatus*, pointed, referring to the shape of apex of anal point.

#### Remarks.

The present new species resembles to *Bryophaenocladius bicolor* Wang, Sæther & Andersen, 2001 in the shape of anal point, but it can be separated from *Bryophaenocladius bicolor* in the following combination of characters in Table 2.

**Table 2.** Differences between *Bryophaenocladius mucronatus* sp.n. and *Bryophaenocladius bicolor* Wang, Sæther & Andersen, 2001.

	*Bryophaenocladius mucronatus* sp. n.	*Bryophaenocladius bicolor* Wang, Sæther & Andersen, 2001
Finger-shaped extension on third palpomere	absent	present
Seta on R_1_	bare	4–5 setae
LR_1_	0.52–0.63, 0.57	0.76–0.82, 0.80
Pseudospurs	present on ta_1_, ta_2_ of mid and hind legs	absent
Crista dorsalis	present	reduced

Female and immature stages unknown.

#### Distribution.

The species was found in Fujian, Sichuan and Zhejiang Provinces (Oriental China).

### Bryophaenocladius parictericus sp. n.

urn:lsid:zoobank.org:act:125AC1CD-0CD6-46B4-AACF-D43C1084EFA6

http://species-id.net/wiki/Bryophaenocladius_parictericus

Figs 5–9

#### Diagnosis.

The male imago can be distinguished from known species of the genus by the following combination of characters: AR 0.52–0.55; third palpomere with apical digitiform projection; Costa extension 115–143, 122 μm long; squama bare; mid tibia comb with 3–7, 5 spines; anal point hyaline, slender with blunt apex; crista dorsalis absent; inferior volsella bubble-shaped, with 8–12, 9 setae.

#### Description.

Male imago (n = 6). Total length 2.65–3.08 2.76 mm. Wing length 1.63–2.48, 2.22 mm. Total length/wing length 1.10–1.46, 1.26. Wing length/length of profemur 2.78–3.19, 3.03.

Coloration. Dark brown.

**Figures 5–9.**
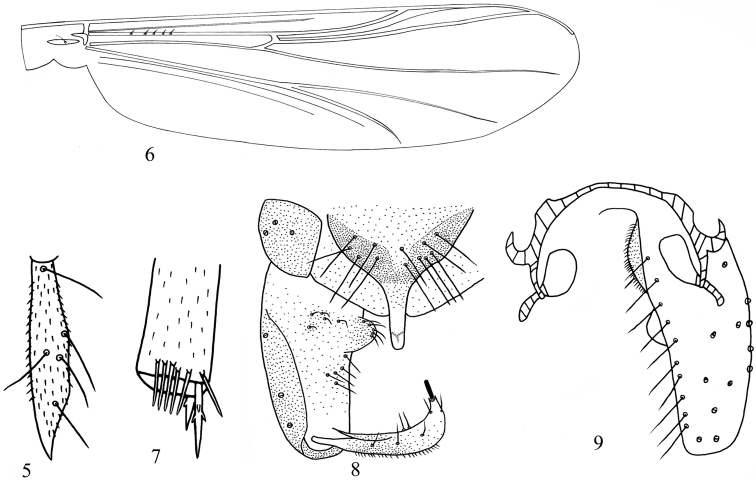
*Bryophaenocladius parictericus* sp. n. **5** third palpomere **6** wing **7** mid tibia **8** hypopygium (dorsal view) **9** hypopygium (ventral view).

Head (Fig. 5). AR 0.52–0.55 (n = 2). Ultimate flagellomere 230–245 (n = 2) μm long. Temporal setae 3–9, 7 including 2–7, 4 inner verticals; 0–4, 2 outer verticals and 0–2, 1 postorbital. Clypeus with 4–7, 5 setae. Tentorium 109–148, 129 μm long, 15–25, 20 μm wide. Stipes 80–100, 90 μm long, 5–8, 6 μm wide. Palpomere lengths (in μm): 16–25, 20; 35–52, 41; 90–143, 114; 42–65, 57; 60–80, 71. L: 5^th^/3^rd^ 0.76–0.80, 0.78. Third palpomere with apical digitiform projection.

Wing (Fig. 6). Anal lobe not developed. Coarse punctation easily visible at 40x magnification. VR 1.02–1.23, 1.17. Costa extension 115–143, 122 μm long. Brachiolum with 1 seate. R with 5–9, 7 setae. Remaining veins bare. Squama bare.

Thorax. Antepronotum with 2–5, 3 lateral setae. Dorsocentrals 8–10, 9; acrostichals 6–7, 7; prealars 2–4, 3. Scutellum with 3–7, 6 setae.

Legs (Fig. 7). Spur of fore tibia 40–58, 48 μm long; spurs of mid tibia 30–42, 38 μm and 21–32, 25 μm long; spurs of hind tibia 40–63, 52 μm and 21–32, 28 μm long. Lateral denticles appressed to main shaft. Mid tibial comb with 3–7, 5 spines; hind tibial comb with 9–14, 12 spines. Mid and hind legs without tarsal pseudospurs. Width at apex of fore tibia 35–45, 40 mm, of mid tibia 33–38, 36 mm, of hind tibia 40–48, 45 mm. Lengths (in μm) and proportions of legs in Table 3.

**Table 3.** Lengths (in μm) and proportions of legs of *Bryophaenocladius parictericus* sp. n.

	_P_1	_P_2	_P_3
fe	510–893, 718	600–914, 798	620–977, 735
ti	710–1134, 916	660–987, 873	770–1260, 994
ta_1_	360–670, 558	320–504, 427	400–683, 611
ta_2_	240–389, 322	170–263, 232	220–315, 282
ta_3_	180–273, 235	140–189, 176	180–284, 233
ta_4_	100–147, 132	70–105, 98	80–126, 115
ta_5_	70–108, 96	70–95, 87	70–105, 95
LR	0.51–0.64, 0.58	0.45–0.51, 0.48	0.52–0.58, 0.54
BV	2.68–2.92, 2.84	3.22–3.68-3.47	3.25–3.35, 3.30
SV	3.24–3.39, 3.31	3.94–4.11, 4.01	3.31–3.48, 3.39
BR	2.14–2.67, 2.33	2.00–2.14, 2.09	1.50–2.11, 2.01

Hypopygium (Figs 8–9). Anal hyaline, slender with blunt apex, 40–55, 48 μm long, 15–20, 18 μm in width. Anal point length/width: 2.22–2.75, 2.51. Tergite IX with 6–13, 9 setae, laterosernite IX with 3–5, 4 setae. Phallapodeme 48–91, 77 μm long. Oral projection of transverse sternapodeme vestigial, 75–96, 85 μm long. Gonocoxite 170–221, 194 μm long. Gonostylus slightly curved, 80–101, 92 μm long. Megaseta 13–21, 18 μm long. Crista dorsalis absent. Inferior volsella bubble-shaped, 18–27, 22 μm long, with 8–12, 9 setae. Virga absent. HR 1.88–2.50, 2.08. HV 2.62–3.48, 3.02.

#### Type materials.

Holotype: ♂ (BDN. K7A22), China, Zhejiang Province: Taizhou City, Xianju County, Shenxianju Scenic Area, 28°42'14"N, 120°36'25"E, 14.iv.2011, Lin XL, sweeping net. Paratypes (5♂♂): 1♂, as Holotype; Sichuan Province: 4♂♂, Yajiang County, 30°01'52"N, 101°00'52"E, 10.vi.1996, 3050 meters above sea level, Wang XH, sweeping net.

#### Etymology.

Named in closing to the species *Bryophaenocladius ictericus* (Meigen, 1830).

#### Remarks.

The present new species resembles to *Bryophaenocladius ictericus* (Meigen, 1830) in the shape of inferior volsella, but it can be separated by following combination of characters in Table 4.

**Table 4.** Differences between *Bryophaenocladius parictericus* sp. n. and *Bryophaenocladius ictericus* (Meigen, 1830)

	*Bryophaenocladius parictericus* sp. n.	*Bryophaenocladius ictericus* (Meigen, 1830)
Antennal ratio (AR)	0.52–0.55	1.19–1.73, 1.56
Finger-shaped extension on third palpomere	present	absent
Length of Costal extension	115–143, 122 μm	64–105, 98 μm
Length of megaseta	13–21, 18 μm	7–14, 11 μm
Gonostylus	bended	straight
Virga	absent	present

Female and immature stages unknown.

#### Distribution.

The species was found in Sichuan and Zhejiang Provinces (Oriental China).

## Supplementary Material

XML Treatment for Bryophaenocladius mucronatus

XML Treatment for Bryophaenocladius parictericus
